# Evaluation of Cardiac Function in Children Undergoing Liver Transplantation

**DOI:** 10.1007/s00246-024-03673-9

**Published:** 2024-11-22

**Authors:** Neha Bansal, Joseph Mahgerefteh, Jacqueline M. Lamour, Debora Kogan-Liberman, Michelle Ovchinsky, Kayla Ganzburg, Nadine Choueiter

**Affiliations:** 1 Division of Pediatric Cardiology, Children’s Hospital at Montefiore, Albert Einstein College of Medicine, 3415 Bainbridge Ave-R1, Bronx, NY 10467 USA; 2 Division of Pediatric Hepatology, Children’s Hospital at Montefiore, Albert Einstein College of Medicine, Bronx, NY USA

**Keywords:** Cirrhotic Cardiomyopathy, Pediatrics, Strain imaging

## Abstract

Cirrhotic cardiomyopathy is a complication of cirrhosis resulting in cardiac dysfunction. It remains poorly characterized in children. The aim of this study was to assess relationship of pre-liver transplant (LT) conventional and novel parameters of biventricular function with post-LT clinical course. This is a retrospective study of pre-LT echocardiograms performed on patients < 18 years of age with cirrhosis at a single center, who received a LT. Demographic, clinical, and echocardiographic data were collected. Speckle tracking echocardiography (STE) analysis was performed by a single observer using TomTec system. Descriptive data were expressed as mean (SD) and number (%). The relationship between clinical data and echocardiographic variables were assessed using Spearman correlation coefficient. Significance was set at < 0.05. Thirty-five patients (median age 6.5; IQR 14.2 years) underwent LT between 2010 and 2020. Pre-LT diagnosis was biliary atresia in 14 (40%) patients and 7 (20%) patients were listed as status 1A/1B. Their median natural pediatric/model end-stage liver disease score was 13 (IQR 9). Their pre-LT echocardiogram showed normal left ventricular systolic (LV) function by ejection fraction and strain parameters. Right ventricular (RV) function was abnormal in 74% of patients as measured by RV GLS (23 ± 3%). There was correlation between echocardiographic parameters with pre-transplant clinical disease and post-operative LT course (length of stay and duration of mechanical ventilation). Children undergoing liver transplant have RV dysfunction as evidenced by abnormal RV GLS on STE. There is echocardiographic parameter correlation between clinical liver disease and post-LT clinical course. This evidence highlights the importance of using novel technology like STE in assessment of children undergoing evaluation for liver transplant.

## Introduction

Cirrhotic cardiomyopathy (CCM) is defined as myocardial dysfunction in patients with cirrhosis [1] and is a hemodynamic complication of portal hypertension characterized by systolic and diastolic cardiac dysfunction [2, 3]. The systolic dysfunction can often be subclinical and may not be apparent by traditional measurements of systolic function such as ejection fraction or fractional shortening. Two-dimensional speckle tracing echocardiography (STE) is a newer technique that measures cardiac fiber shortening, and has been shown to reliably detect early left ventricular systolic dysfunction in adult and pediatric patients before overt changes in traditional echocardiographic measures appear [4–7]. In pediatric patients with anthracycline-induced cardiomyopathy [6], diabetes mellitus [8], and muscular dystrophy [9], STE may be superior to conventional measurements for detection of early myocardial dysfunction.

In adults with cirrhosis, impaired LV myocardial contractility is noted by STE and it improves following liver transplantation (LT) [1]. The 2005 World Congress of Gastroenterology criteria for CCM were revised in 2020 to incorporate longitudinal strain [10]. However, due to lack of an established pediatric definition, CCM in children remains poorly characterized [11]. The ability to detect early dysfunction could lead to earlier medical or procedural therapy and potentially prevent overt cirrhotic cardiomyopathy. The hypotheses of this study were (i) biventricular systolic function as measured by STE is worse in children with more severe liver disease and (ii) predict worse post-liver transplantation (LT) clinical course. The aims of this study were to (i) assess prevalence of biventricular dysfunction by STE in children with cirrhosis, (ii) assess the relationship of conventional and STE echocardiographic parameters of biventricular function with clinical disease score in this patient population, and (iii) assess relationship of pre-transplant echocardiographic parameters of biventricular function with post-LT clinical course.

## Methods

This was a retrospective observational study of all patients with cirrhosis < 18 years of age, who received a liver transplant at the Children’s Hospital at Montefiore (CHAM) from 1/1/2010 to 1/31/2020. The study was approved by the Institutional Review Board at the Albert Einstein College of Medicine. Patients with known history of cardiomyopathy, congenital heart disease (including Alagille patients with significant valvar disease), and systemic conditions affecting cardiac function such as lupus or history of cardiopulmonary resuscitation were excluded from analysis. Patients who had LT for other indications (such as hepatoblastoma, various metabolic disorders, acute liver failure) and did not have cirrhosis were also excluded. Data including demographic data (age, gender), clinical data, details of liver disease, and laboratory measurements were extracted from the electronic medical record (Table 1). The Pediatric End-Stage Liver Disease (PELD) and MELD (Model for End-Stage Liver Disease) are prioritization scores that reflect the risk of death on the transplant waitlist within a 90-day period, and are used to allocate livers for transplantation [12]. Patients who had echocardiograms performed at institutions outside of CHAM were excluded from this study. Echocardiograms were performed using Philips Sono IE 33 machine (Philips, Andover MA). A single reader (NB) blinded to the clinical data performed STE.

**Table 1:** Demographic and clinical parameters in patients listed for liver transplantation

Variable Mean ± SD/*n* (%)	*N* = 35
Age at Transplant (years)	7.5 ± 7
Weight (Kg)	27.9 ± 26.4
BMI	19.8 ± 8.9
Female Gender	21 (60%)
Diagnosis Biliary Atresia Autoimmune Hepatitis Alagille Syndrome Others	14 (40%) 4 (11.4%) 3 (8.5%) 14 (40%)
Listed as Status 1A/1B	7 (20%)
Natural PELD/MELD score	13.1 ± 9.9
Ascites	21 (60%)
Varices on Scope Yes No No scope	17 (48.6%) 2 16 (45.7%)
GI Bleed	10 (28.6%)
Supplemental enteral nutrition	16 (45.7%)
Supplemental parenteral nutrition	7 (20%)
Admitted to PICU pre transplant	5 (14.3%)
Mean albumin levels	3.66 ± 0.7
Mean Prothrombin Time	16.58 ± 6.3
Mean INR	1.5 ± 0.6
Mean BUN	11.1 ± 3.8
Mean creatinine	0.4 ± 0.2
Living Donor	11 (31.4%)
Whole Graft Transplant	16 (45.7%)
Post-Op inotropic requirement	0 (0%)
Post-Op Biliary Complications	9 (25.7%)
Portal vein thrombosis	1 (2.9%)
Hepatic artery thrombosis	0 (0%)

**Fig. 1:**
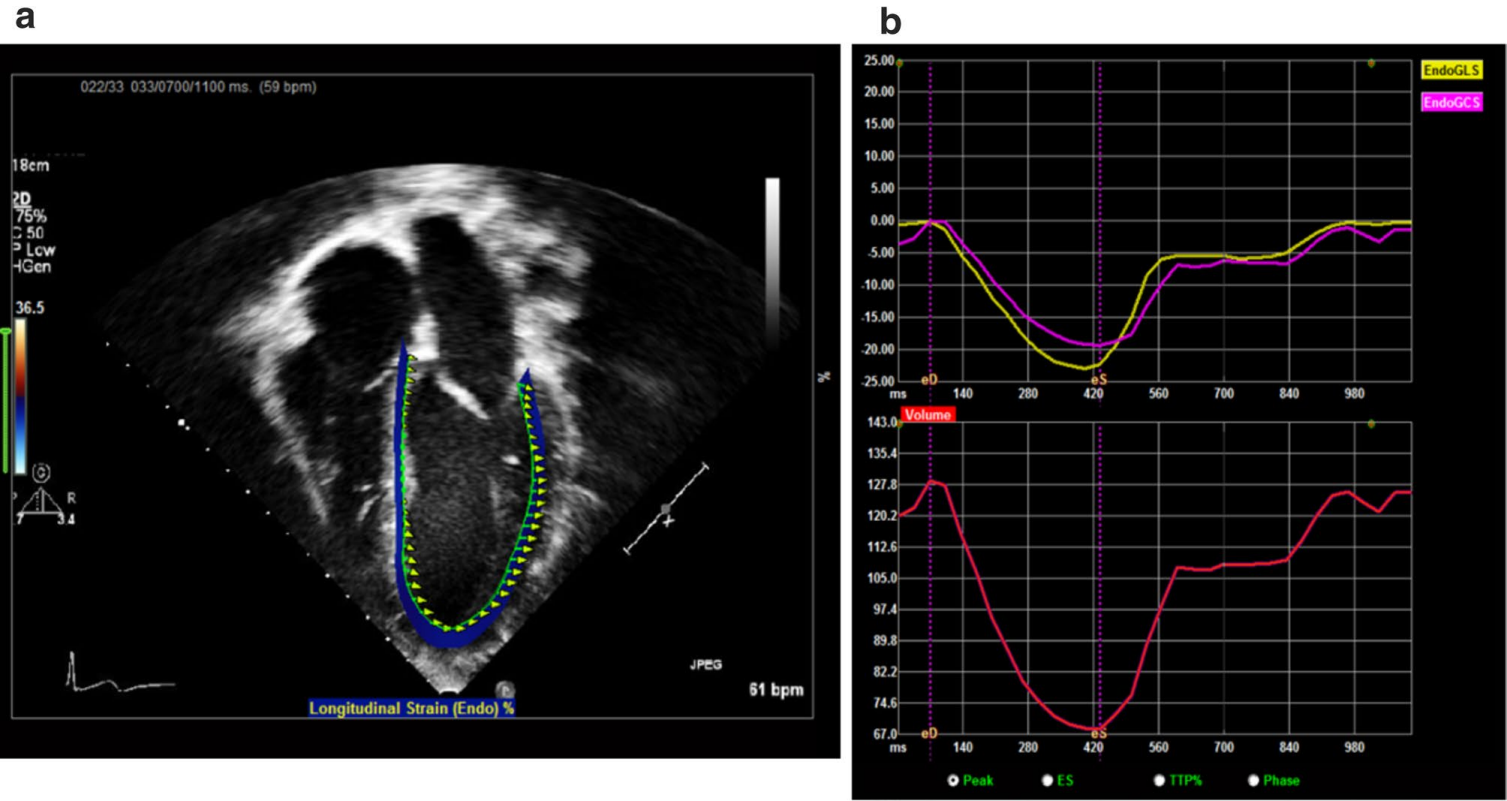
Tracings of peak endocardial LV strain using TomTec Imaging System

STE was performed offline on previously completed echocardiograms using validated, vendor-independent, 2D STE software (2D Cardiac Performance Analysis v1.1; TomTec Imaging Systems, Unterschleissheim, Germany). Strain was measured by tracing the endocardial border of the left and right ventricle at the initial frame with the best endocardial border definition across the maximal number of segments in an apical four-chamber view only (Fig. 1). Circumferential strain was measured in parasternal short-axis view. The average value of all segments, defined as the global peak longitudinal systolic strain of the left ventricle (GLS), included longitudinal global strain (%) and strain rate (SR) (1/sec). For strain and SR, a lower absolute value indicates worse ventricular function. According to the reference values published in a study of 1443 healthy children, the normal mean values of GLS from apical four-chamber view varied from − 15.1% to − 24.8% (mean − 20.4, 95% CI − 19.8% to − 21.7%) and the normal mean values of systolic SR varied from − 0.41 to − 2.59 (mean − 1.20, 95% CI − 0.96 to − 1.44) [13]. Similarly, the reference values of normal mean RV GLS in children is from − 20.80% to − 34.10% (mean − 29.03, 95% CI − 31.52 to − 26.54 [14].

Standard methodology was used for measuring conventional echocardiographic parameters. A conventional M-mode echocardiogram was used to calculate shortening fraction using standard techniques. Ejection fraction was calculated using the 5/6 area-length method using the formula V = 5/6*area*length. The right ventricular fractional area change (FAC) was calculated by tracing the endocardial boarders in end-diastole and end-systole on the apical 4-chamber view and calculating the percentage change in area. Tricuspid annular plane systolic excursion (TAPSE) was measured using M-mode echocardiography in the apical four-chamber view. TAPSE was measured as the total displacement of the tricuspid annulus (cm) from end-diastole to end-systole and a value of < 1.8 cm was considered abnormal [15]. Fractional shortening was calculated using M-mode in the parasternal long-axis window. The RV fractional area change (FAC) was obtained from the apical four-chamber view, and was calculated as the difference in end-diastolic area and end-systolic area divided by the end-diastolic area.

Diastolic parameters were measured and given the limitations of these measurements in pediatrics as shown in previous studies [16]; the three widely accepted measurements were mitral inflow early-to-late diastolic flow (E/A) ratio (> 2 or < 0.8 considered abnormal), mitral E-to-mean e′ ratio (> 10 considered abnormal), and left atrial volume (by the area-length method) indexed to body surface area (LAVi) with > 34 ml/m2 considered abnormal. All measurements were performed offline from standard views, according to current ASE recommendations [17].

### Statistical Analysis

Descriptive statistics were reported as mean ± standard deviation or median (interquartile range (IQR)). The relationship between echocardiographic parameters and clinical variables was assessed using Spearman correlation coefficient. A p value of < 0.05 was considered statistically significant. The software SPSS 17.0 for windows was used for the analysis.

## Results

### Demographic and Clinical Data

A total of 35 patients received a liver transplant with a median age at transplant of 6.5 (IQR 14.2) years and mean BMI of 19.8 ± 8.9 kg/m^2^. The majority of the patients were female 21 (60%). Fourteen (40%) patients had a diagnosis of biliary atresia, 4 (11.4%) patients had autoimmune hepatitis, and 3 patients had Alagille Syndrome. The median natural Model for End-Stage Liver Disease (MELD) or Pediatric End-Stage Liver Disease (PELD) score was 13 (IQR 9). Seven (20%) patients were listed status 1A/1B for transplantation.

Twenty-one (60%) patients had a history of ascites, 17 (48.5%) patients had varices on endoscopy, and 10 (28.6%) patients had a history of gastrointestinal (GI) bleeding. Sixteen (45.7%) patients were on supplemental enteral nutrition and 7 (20%) were on supplemental parenteral nutrition. The mean albumin levels were 3.7 ± 0.7 and mean INR for the cohort was 1.5 ± 0.6 with 9/35 (25%) having elevated INR values > 1.5. All patients had normal BUN and creatinine values.

Eleven (31.4%) patients had living donor transplantation and 16 (45.7%) had whole graft transplantation. Post LT, the median duration of mechanical ventilation was 1 day (IQR 0, 1.9), the median hospital length of stay (LOS) was 14 days (IQR 7, 18), and the ICU LOS was 5 days (IQR 3, 10.5). No post-operative inotropic infusions were needed. Post LT, 9 patients had biliary complications, 1 patient had portal vein thrombosis, and none had hepatic artery thrombosis. The basic demographic and clinical parameters are summarized in Table 1.

### Echocardiographic Data

The pre-liver transplant echocardiogram for all patients showed normal LV ejection fraction (EF) (> 55%) with mean LVEF 63.15 ± 4.5% **(**Table 2**)**. The right ventricular (RV) fractional area change (> 35%) was normal in all patients (mean 41.04 ± 4.6%). The tricuspid annular plane systolic excursion (TAPSE) was abnormal (< 1.8 cm) in 15 (42.8%) patients with mean value being 1.8 ± 0.6 cm [16].

**Table 2:** Echocardiographic parameters of the cohort listed for liver transplantation

Variable *N* = 35	Mean ± SD *n* (%)
TAPSE (cm)	1.8 ± 0.6
Patients with Abnormal TAPSE	17 (48.5%)
LV EF (%)	63.2 ± 4.5
LV Mass index (g)	59.66 ± 17.8
LAVI (ml^2^)	28.9 ± 10.1
Patients with LAVI > 34	4/35
RV FAC (%)	41 ± 4.6
LV GLS (%)	− 22.2 ± 2.7
Patients with Abnormal LV GLS	1/35
LV GLS SR (s^−1^)	− 1.15 ± 0.2
LV GCS (%)	− 25.7 ± 4
LV GCS SR (s^−1^)	− 1.3 ± 0.4
RV GLS (%)	− 22.9 ± 2.7
Patients with abnormal RV GLS	26/35 (74%)
RV GLS SR (s^−1^)	− 1.2 ± 0.2
MV E/A	1.65 ± 0.5
MV E/e′ lateral (n = 26)	6.97 ± 2.6
MV E/e′ medial (n = 26)	9.03 ± 2.3
Evidence of Diastolic Dysfunction	13/26 (50%)

Parameters of left ventricular diastolic dysfunction (DD) were measured in 26/35 patients of which 13/26 (50%) had evidence of DD. Five patients had an E/A ratio > 2, none had E/A ratio < 0.8, and 5 had abnormal E/e′ ratios (> 10) of the mitral annulus (either lateral or medial). The mean left atrial volume index (LAVI) was 28.9 ± 10.1 ml/m^2^ and 4 patients had LAVI > 34 ml/m^2^.

The mean LV global longitudinal strain (GLS) was − 22.2 ± 2.8%. Of the 35 patients, only one patient had abnormal LV GLS (< 18%) [12]. The average GLS strain rate was − 1.15 ± 0.2 /sec. The LV circumferential strain was normal in all patients with mean values being − 25.7 ± 4% and strain rate being − 1.26 ± 0.4. The RV GLS was abnormal (values less than − 25% using previously published normal range) in 26/35 (74%) of the patients with the mean values being 22.9 ± 2.7% [14].

Using Spearman correlation test, a statistically significant correlation was found between echocardiographic parameters and pre-transplant clinical factors as well as post-LT clinical course and complications (Table 3). The pre-transplant clinical features, e.g., age at transplant and history of GI bleed, correlated with LV GLS (*r* = 0.35 *p* = 0.04 & *r* = 0.41 *p* = 0.013). RV GLS did not correlate with any clinical features pre or post LT. TAPSE and evidence of diastolic dysfunction correlated with markers of worse liver disease such as decompensated portal hypertension, need for admission to PICU pre LT, need for supplemental enteral nutrition, PELD/MELD scores, prothrombin time, and INR. Post LT, duration of mechanical ventilation, post-transplant ICU stay, and total length of stay also correlated with TAPSE and diastolic dysfunction.

**Table 3:** Correlation of echocardiographic parameters with pre-and post-liver transplant clinical parameters

Spearman Correlation	LV EF		LV GLS		RV FAC		RV GLS		TAPSE		MV E/A		E/e′ (Lateral)		E/e′ (Medial)	
	r	*p* value	r	*p* value	r	*p* value	r	*p* value	r	*p* value	r	*p* value	r	*p* value	r	*p* value
Age at Transplant	0.03	0.8	**0.35**	**0.041**	− 0.05	0.7	− 0.04	0.8	**0.8**	**0.0001**	**0.63**	**0.0001**	− 0.33	0.1	− **0.65**	**0.0001**
Decompensated portal HTN	− 0.03	0.8	− 0.14	0.4	− 0.32	0.05	0.175	0.3	− **0.45**	**0.007**	− 0.39	0.025	**0.52**	**0.007**	**0.44**	**0.025**
GI Bleed	− 0.081	0.6	**0.41**	**0.013**	0.09	0.6	− 0.29	0.08	0.038	0.8	0.19	0.3	0.15	0.5	0.16	0.4
Ascites	− 0.05	0.7	− 0.09	0.6	− 0.19	0.3	− 0.09	0.6	**0.035**	**0.037**	0.26	0.1	− 0.26	0.19	− 0.077	0.7
Admission to PICU	0.23	0.19	0.008	0.9	− 0.28	0.1	− 0.24	0.16	**0.59**	**0.0001**	**0.35**	**0.045**	− 0.29	0.1	− 0.38	0.052
Supplemental Enteral nutrition	0.09	0.6	− 0.03	0.9	0.014	0.9	− 0.03	0.8	**0.76**	**0.0001**	**0.38**	**0.029**	− 0.35	0.08	− **0.6**	**0.001**
Prothrombin Time	0.17	0.3	0.28	0.1	0.03	0.8	0.24	0.17	− **0.335**	**0.049**	− **0.39**	**0.02**	**0.41**	**0.036**	**0.43**	**0.029**
INR	0.13	0.46	0.18	0.3	− 0.11	0.5	0.13	0.45	− **0.402**	**0.017**	− 0.22	0.2	**0.47**	**0.016**	**0.54**	**0.004**
Natural PELD/MELD score	0.036	0.8	− 0.01	0.9	− 0.3	0.056	0.17	0.3	− **0.45**	**0.007**	− **0.39**	**0.025**	**0.516**	**0.007**	**0.439**	**0.025**
Post-op mechanical ventilation	0.03	0.86	− 0.11	0.5	0.2	0.24	0.001	0.9	− **0.54**	**0.001**	− **0.35**	**0.047**	0.18	0.3	**0.47**	**0.017**
Post-op ICU stay	0.07	0.7	0.08	0.6	0.26	0.13	− 0.02	0.8	− **0.52**	**0.001**	− **0.48**	**0.005**	0.32	0.1	**0.49**	**0.012**
Post-Op Hospital LOS	− 1.04	0.6	− 0.05	0.7	0.14	0.4	− 0.08	0.6	− **0.7**	**0.0001**	− **0.36**	**0.039**	**0.4**	**0.045**	**0.54**	**0.005**
Post-op biliary complication	0.14	0.4	0.08	0.6	− **0.38**	**0.025**	0.11	0.5	0.042	0.8	− 0.018	0.9	0.178	0.4	− 0.005	0.9
Portal vein thrombosis	− 0.12	0.49	0.27	0.1	− 0.22	0.2	− 0.03	0.8	0.22	0.2	0.13	04	− 0.3	0.12	− 0.17	0.4

Bold indicates statistically significant

In addition, a strong correlation was found between pre-LT clinical features, such as age at transplant, PELD/MELD scores, presence of ascites/varices, and need for supplemental nutrition, and post-LT clinical findings, including the need for mechanical ventilation, post-op ICU, and total length of stay **(**Table 4**).** However, post-LT complications like biliary atresia and portal vein thrombosis did not correlate with pre-LT clinical parameters.

**Table 4:** Correlation of pre-liver transplant clinical parameters with post-transplant clinical course

Spearman Correlation	Age at Transplant		PELD/MELD Score		Ascites		Supplemental Enteral Nutrition		Varices on Scope		Prothrombin Time		Creatinine	
	r	*p* value	r	*p* value	r	*p* value	r	*p* value	r	*p* value	r	*p* value	r	*p* value
Post-op mechanical ventilation	− **0.63**	**0.001**	0.17	0.3	− **0.346**	**0.042**	− **0.47**	**0.004**	**0.45**	**0.007**	**0.4**	**0.016**	− **0.553**	**0.001**
Post-op ICU stay	− **0.69**	**0.001**	**0.39**	**0.021**	− **0.53**	**0.001**	− **0.46**	**0.005**	0.29	0.09	**0.46**	**0.005**	− **0.613**	**0.0001**
Post-Op Hospital LOS	− **0.57**	**0.001**	**0.34**	**0.046**	− **0.42**	**0.011**	− **0.57**	**0.0001**	0.3	0.054	**0.36**	**0.035**	− **0.59**	**0.0001**
Post-op biliary complication	− 0.16	0.3	0.32	0.057	− 0.053	0.7	− 0.15	0.9	− 0.08	0.6	0.28	0.1	0.2	0.24
Portal vein thrombosis	0.25	0.14	− 0.094	0.59	0.14	0.4	0.18	0.3	− 0.18	0.3	− 0.13	0.4	0.26	0.12

Bold indicates statistically significant

## Discussion

Cirrhotic cardiomyopathy is characterized by a hyperdynamic circulatory state with high cardiac output at rest due to a low systemic vascular resistance [18]. It remains a poorly described entity in pediatrics. It carries significant mortality and morbidity including increased incidence of perioperative complications and graft rejection [19, 20]. Our study assesses conventional and strain echocardiographic parameters of ventricular function in pediatric patients with cirrhosis who are listed for liver transplantation.

We found that an overwhelming 74% of our cohort had abnormally low RV GLS values prior to liver transplantation when RV FAC was normal. However, RV GLS did not seem to correlate with post-LT clinical course. There are several proposed mechanisms of RV dysfunction in patients with liver cirrhosis. These include decreased cardiac responsiveness (chronotropic and inotropic incompetence) through the defect in cardiac β-adrenergic receptor signaling, higher production of cardiac depressant substances such as nitric oxide and carbon monoxide, increased concentration of inflammatory cytokines such as interleukin-8, Interleukin-6, Interleukin-1b, TNF-α, as well as elevated catecholamine levels as a result of sympathetic over activity [21]. In addition, elevated pulmonary arterial pressure may further contribute to the dysfunction [1]. Our study also found that TAPSE, a parameter of RV function, correlates with post-transplant clinical course including post-transplantation hospital length of stay, length of ICU stay, and duration of mechanical ventilation, thus highlighting the importance of assessment of RV in patients with cirrhosis.

Overt systolic dysfunction has been observed in only 2% of adult liver transplant candidates [22]. This is similar to our data that almost all patients had normal LV function as measured by EF and only one patient had abnormal LV GLS strain. Previous pediatric studies have shown that systolic strain was impaired in patients with cirrhosis [1]. LV function by EF or strain did not correlate with post-LT clinical course. However, none of the patients in our cohort required inotropes post LT, which might affect the results of our study.

Our study found a significant burden of diastolic dysfunction (DD) in pediatric patients with almost half of the patients having DD on the pre-LT echocardiogram. Increasing numbers of recent studies have emphasized the importance of LVDF in predicting poor early and late outcomes after LT [18, 23, 24]. Raevens et al. found incidence of DD in 43% in 173 adult liver transplant candidates [22]. Our study showed almost 50% of patients had DD. This is similar to the existing evidence [25, 26]. In our cohort, the markers of DD in pediatric patients correlated with a longer post-LT hospital length of stay, length of ICU stay, and duration of mechanical ventilation. In adults, a combination of systolic and diastolic dysfunction has been shown to predict survival outcomes in patients undergoing LT [18]. Thus, it becomes important to evaluate these parameters in patients. Our small sample size did not allow for assessment of mortality outcome, as no mortalities occurred in this cohort.

In our study, there was no correlation between LV systolic function and severity of disease. However, TAPSE and parameters of DD correlated with severity of liver disease as seen by natural PELD/MELD score, prothrombin time, INR, and decompensated portal hypertension. Evidence of this is in contrast to a study of patients with cirrhosis where no significant association was found between echocardiographic changes and Child–Pugh score [27].

Pediatric liver waitlist morbidity and mortality are high overall but unpredictable in individual cases, resulting in poor stratification and general dissatisfaction with the current system of liver allocation in infants and children [28]. In the absence of improved organ availability, new objective parameters for children are needed. The echocardiographic parameters and data from our study can be used to model further efforts in the stratification of cardiovascular risk and may change treatment decisions.

There are limitations to this study. It is a single-center retrospective analysis with a limited sample size and a significant heterogeneity in primary diagnosis. STE is dependent on image quality and strain values are subject to error, though they were performed in a uniform and standardized manner. However, our site has previously published data on strain in patients with PVCs with an inter-rater reliability value of 0.87 demonstrating that the values are reproducible [7]. The measurements of LV and RV function by the area-length method and fractional area shortening have their inherent assumptions and limitations. Only a single echocardiogram was reported for each patient, so the authors cannot extrapolate the data longitudinally. The retrospective nature of the study did not allow for apical 2-chamber and 3-chamber evaluations. We also used absolute values for TAPSE and not the TAPSE z scores as the z scores have not been validated in this cohort. In addition, clinically many of our patients had low PELD/MELD scores (range is from 6 to 40), indicating less severe liver disease, which may affect our findings and limit the application to all pediatric cirrhosis patients. Lastly, we recognize that this is a small study with confounding factors and it is possible that other factors may have contributed to the cardiac findings and the results only address association and not causation. Further larger studies are warranted to build on the existing findings.

## Conclusion

Cirrhotic children undergoing liver transplantation seem to have preserved LV systolic function but subclinical RV dysfunction and diastolic function. There is echocardiographic correlation with severity of liver disease as well as with immediate post-transplant clinical course. This evidence highlights the importance of using novel technology like STE in assessment of children undergoing evaluation for liver transplant. Conventional and STE parameters of ventricular function can be used to further stratify disease severity in cirrhotic children and may change treatment decisions especially when pediatric liver waitlist morbidity and mortality are overall high.

## Data Availability

No datasets were generated or analysed during the current study.
